# Supplementary material for: Auricular Vagus Neuromodulation—A Systematic Review on Quality of Evidence and Clinical Effects
Source: Front Neurosci. 2021 Apr 30;15:664740. doi: 10.3389/fnins.2021.664740 (PMC8120162; doi:10.3389/fnins.2021.664740)
Supplement: Supplementary file 3 [file Data_Sheet_1.ZIP › Supplementary 9 - Individual RoB Rubrics/Burger (2019a) Parallel (Individually Randomised) - Per Protocol Analysis.docx]

# The RoB 2.0 tool (individually randomized, parallel group trials)

| **Assessor name/initials** | JM |
| --- | --- |
| **Study ID and/or reference(s)** | Burger (2019a) |

**Study design**

| 🗹 | Randomized parallel group trial |
| --- | --- |
| □ | Cluster-randomized trial |
| □ | Randomized cross-over or other matched design |

| **Specify which outcome is being assessed for risk of bias** | Number of negative thought intrusions |
| --- | --- |

| **Specify the numerical result being assessed.** In case of multiple alternative analyses being presented, specify the numeric result (e.g. RR = 1.52 (95% CI 0.83 to 2.77) and/or a reference (e.g. to a table, figure or paragraph) that uniquely defines the result being assessed. | tVNS showed significant reduction compared to sham (1.0 SD 1.2 vs 1.5 SD 1.4, p=0.037) in pre-worry induction period. However, tVNS yielded slightly higher numbers of negative thought intrusion after worry induction and post-worry period (though these were not reported as significant, p=0.83). |
| --- | --- |

**Is your aim for this study…?**

| □ | to assess the effect of *assignment to intervention* |
| --- | --- |
| 🗹 | to assess the effect of *starting and adhering to intervention* |

**Which of the following sources have you obtained to help inform your risk of bias judgements (tick as many as apply)?**

🗹 Journal article(s) with results of the trial

□ Trial protocol

□ Statistical analysis plan (SAP)

□ Non-commercial trial registry record (e.g. ClinicalTrials.gov record)

□ Company-owned trial registry record (e.g. GSK Clinical Study Register record)

□ “Grey literature” (e.g. unpublished thesis)

□ Conference abstract(s) about the trial

□ Regulatory document (e.g. Clinical Study Report, Drug Approval Package)

□ Research ethics application

□ Grant database summary (e.g. NIH RePORTER, Research Councils UK Gateway to Research)

□ Personal communication with trialist

□ Personal communication with the sponsor

## Risk of bias assessment for a parallel group trial with interest in the effect of starting and adhering to intervention

| **Domain** | **Signalling questions** | **Response options** | **Description/Support for judgement** |
| --- | --- | --- | --- |
| **Bias arising from the randomization process** | 1.1 Was the allocation sequence random? | Y | Subjects were randomly allocated  No reason to suspect pre-emptive revealing of the allocation sequence |
|  | 1.2 Was the allocation sequence concealed until participants were recruited and assigned to interventions? | PY |  |
|  | 1.3 Were there baseline imbalances that suggest a problem with the randomization process? | PN | No reason to suspect baseline imbalances |
|  | **Risk of bias judgement** | Low | From algorithm |
|  | Optional: What is the predicted direction of bias arising from the randomization process? | Unpredictable |  |
| **Bias due to deviations from intended interventions** | 2.1. Were participants aware of their assigned intervention during the trial? | PN | Decent sham, unlikely that subjects figured out their allocation  Not stated but likely yes |
|  | 2.2. Were carers and trial personnel aware of participants' assigned intervention during the trial? | NI |  |
|  | 2.3. If Y/PY/NI to 2.1 or 2.2: Were important co-interventions balanced across intervention groups? | PY | Interventions were well-balanced |
|  | 2.4. Was the intervention implemented successfully? | Y | No reported issues with intervention implementation |
|  | 2.5. Did study participants adhere to the assigned intervention regimen? | PY | No reported divergence of participants from assigned intervention |
|  | 2.6. If N/PN/NI to 2.3, 2.4 or 2.5: Was an appropriate analysis used to estimate the effect of starting and adhering to the intervention? | NA |  |
|  | **Risk of bias judgement** | Low | From algorithm. |
|  | Optional: What is the predicted direction of bias due to deviations from intended interventions? | Blank |  |
| **Bias due to missing outcome data** | 3.1 Were outcome data available for all, or nearly all, participants randomized? | Y | No reason to suspect missing outcome data |
|  | 3.2 If N/PN/NI to 3.1: Are the proportions of missing outcome data and reasons for missing outcome data similar across intervention groups? | NA |  |
|  | 3.3 If N/PN/NI to 3.1: Is there evidence that results were robust to the presence of missing outcome data? | NA |  |
|  | **Risk of bias judgement** | Low | From algorithm |
|  | Optional: What is the predicted direction of bias due to missing outcome data? | Blank |  |
| **Bias in measurement of the outcome** | 4.1 Were outcome assessors aware of the intervention received by study participants? | NI | State of blind not reported |
|  | 4.2 If Y/PY/NI to 4.1: Was the assessment of the outcome likely to be influenced by knowledge of intervention received? | PN | Physiological or patient-reported measurements, so bias affect unlikely |
|  | **Risk of bias judgement** | Low | From algorithm |
|  | Optional: What is the predicted direction of bias due to measurement of the outcome? | Blank |  |
| **Bias in selection of the reported result** | Are the reported outcome data likely to have been selected, on the basis of the results, from... |  |  |
|  | 5.1. ... multiple outcome measurements (e.g. scales, definitions, time points) within the outcome domain? | PN | Multiple time points and metrics, but specified well in pre-registration. |
|  | 5.2 ... multiple analyses of the data? | NI | Many analyses were performed, analysis intentions not specified |
|  | **Risk of bias judgement** | Some concerns | From algorithm |
|  | Optional: What is the predicted direction of bias due to selection of the reported result? | Blank |  |
| **Overall bias** | **Risk of bias judgement** | Some concerns | well executed study |
|  | Optional:  What is the overall predicted direction of bias for this outcome? | Unpredictable |  |
